# HBZ impedes the Menin function and up-regulates the transcription of the hTERT gene in leukemic cells

**DOI:** 10.1186/1742-4690-8-S1-A182

**Published:** 2011-06-06

**Authors:** Malgorzata Borowiak, Anne-Sophie Kuhlmann, Sophie Girard, Louis Gazzolo, Madeleine Duc Dodon

**Affiliations:** 1 Virologie Humaine, INSERM-U758, Ecole Normale Supérieure de Lyon, IFR 128 BioSciences Lyon-Gerland, Lyon, Cedex 07,69364, France

## 

Leukemic cells from Adult T-cell leukemia (ATL) patients display elevated telomerase activity, resulting mainly from transcriptional up-regulation of the human telomerase catalytic subunit (hTERT). We have previously shown that HBZ (HTLV-1 bZip) protein cooperates with JunD transcription factor to enhance hTERT expression after JunD anchoring to Sp1 bound to Sp1 sites within the hTERT proximal promoter. In normal somatic cells, telomerase expression is negatively regulated by tumor suppressor gene products, such as Menin, encoded by the multiple endocrine neoplasia type 1 (MEN-1) gene. Interestingly, the interaction of Menin with JunD has been shown to repress its transcriptional activity.

We report here that, in HBZ-expressing cells, Menin and HBZ exert opposite effects on JunD-mediated regulation of hTERT transcription. Chromatin immuno-precipitation as well as functional assays demonstrate that this antagonism is linked to the recruitment of p300 by HBZ and HDACs by Menin. Furthermore, knock-down of Menin in the ATL Tl-Om1 (only expressing HBZ) cells results in an increase of hTERT expression and of telomerase activity, whereas a knock-down of HBZ exerts opposite effects. Interestingly, primary leukemic cells isolated from ATL patients that express high amounts of hTERT transcripts, are also characterized by an elevated expression of HBZ and MEN-1 genes. Thus, in leukemic cells, HBZ behaves as a key factor impeding the Menin function and sustaining hTERT transcription. These findings underline the critical role of HBZ as a tumor-promoting protein during the development of the HTLV-1-induced leukemogenic process.

